# Is MR Spectroscopy Really the Best MR-Based Method for the Evaluation of Fatty Liver in Diabetic Patients in Clinical Practice?

**DOI:** 10.1371/journal.pone.0112574

**Published:** 2014-11-26

**Authors:** Daniella Braz Parente, Rosana Souza Rodrigues, Fernando Fernandes Paiva, Jaime Araújo Oliveira Neto, Lilian Machado-Silva, Valeria Lanzoni, Carlos Frederico Ferreira Campos, Antonio Luis Eiras-Araujo, Pedro Emmanuel Alvarenga Americano do Brasil, Philippe Garteiser, Marilia de Brito Gomes, Renata de Mello Perez

**Affiliations:** 1 D′Or Institute for Research and Education, Rio de Janeiro, Brazil; 2 Federal University of Rio de Janeiro, Rio de Janeiro, Brazil; 3 University of the State of Rio de Janeiro, Rio de Janeiro, Brazil; 4 Institute of Physics of São Carlos, University of São Paulo, São Carlos, Brazil; 5 Federal University of São Paulo, São Paulo, Brazil; 6 Université Paris Diderot Sorbonne, Paris, France; University of Pisa, Italy

## Abstract

**Objective:**

To investigate if magnetic resonance spectroscopy (MRS) is the best Magnetic Resonance (MR)-based method when compared to gradient-echo magnetic resonance imaging (MRI) for the detection and quantification of liver steatosis in diabetic patients in the clinical practice using liver biopsy as the reference standard, and to assess the influence of steatohepatitis and fibrosis on liver fat quantification.

**Methods:**

Institutional approval and patient consent were obtained for this prospective study. Seventy-three patients with type 2 diabetes (60 women and 13 men; mean age, 54±9 years) underwent MRI and MRS at 3.0 T. The liver fat fraction was calculated from triple- and multi-echo gradient-echo sequences, and MRS data. Liver specimens were obtained in all patients. The accuracy for liver fat detection was estimated by receiver operator characteristic (ROC) analysis, and the correlation between fat quantification by imaging and histolopathology was analyzed by Spearman's correlation coefficients.

**Results:**

The prevalence of hepatic steatosis was 92%. All gradient-echo MRI and MRS findings strongly correlated with biopsy findings (triple-echo, rho = 0.819; multi-echo, rho = 0.773; MRS, rho = 0.767). Areas under the ROC curves to detect mild, moderate, and severe steatosis were: triple-echo sequences, 0.961, 0.975, and 0.962; multi-echo sequences, 0.878, 0.979, and 0.961; and MRS, 0.981, 0.980, and 0.954. The thresholds for mild, moderate, and severe steatosis were: triple-echo sequences, 4.09, 9.34, and 12.34, multi-echo sequences, 7.53, 11.75, and 15.08, and MRS, 1.71, 11.69, and 14.91. Quantification was not significantly influenced by steatohepatitis or fibrosis.

**Conclusions:**

Liver fat quantification by MR methods strongly correlates with histopathology. Due to the wide availability and easier post-processing, gradient-echo sequences may represent the best imaging method for the detection and quantification of liver fat fraction in diabetic patients in the clinical practice.

## Introduction

Non-alcoholic fatty liver disease (NAFLD) affects 10–30% of the general population across all ethnicities and age groups [1]–[5]. Obesity and diabetes are the primary risk factors. Worldwide, the prevalence of obesity, diabetes, and fatty liver is rising [1], [3]–[5]. NAFLD is a clinicopathologic syndrome with a wide spectrum of histological abnormalities and clinical outcomes [1], [2], [4], . While some patients have isolated steatosis, others have non-alcoholic steatohepatitis (NASH), which can progress to cirrhosis, and are at increased risk for hepatocellular carcinoma [1], [2], [4], [6], [7]. Today, NAFLD is the third most common indication for liver transplantation in the United States and is on a trajectory to become the most common indication [8]. Therefore, early diagnosis is important for appropriate treatments and to prevent progression.

The prevalence of NAFLD in diabetic patients is approximately 70% [9]–[12]. Type 2 diabetes is an important risk factor for NAFLD and the disease follows a more aggressive course in these patients with necroinflammation and fibrosis [13]–[17]. The prevalence of NASH in diabetic patients is not well established [18], but has been estimated to be between 22% and 88% [11], [19]. Diabetic patients commonly have comorbidities (e.g., obesity or coronary heart disease) and may be at higher risk of complications during liver biopsy [20]. Therefore, noninvasive methods to diagnose NAFLD, quantify liver fat, stage disease severity, and monitor patients over time is important.

Several imaging methods have been used in the diagnosis of NAFLD. Ultrasonography (US) is the most common and accurately detects moderate and severe NAFLD, but it is not sensitive to mild steatosis [21]–[23]. Interobserver agreement for the severity of NAFLD can be as low as 55% [24]. Therefore, US does not allow precise fat liver quantification for patient follow-up. Computed tomography (CT) is also limited for the detection of mild steatosis and ionizing radiation prevents its use for long-term monitoring [21]–[23], [25]. Magnetic resonance imaging (MRI) and magnetic resonance spectroscopy (MRS) are considered excellent imaging methods for the noninvasive detection and quantification of liver fat [23], [26]–[28]. However, the accuracy of MRI and MRS in the detection and quantification of liver fat fraction, the thresholds for mild, moderate and severe steatosis, and the influence of steatohepatitis and fibrosis on the quantification of liver fat fraction are still unknown. Therefore, the goal of this study was to assess the diagnostic performance of triple- and multi-echo MRI, and 1H spectroscopy in the evaluation of hepatic steatosis, to determine the thresholds for different steatosis grades, and to assess if steatohepatitis and/or fibrosis changes MR quantification of liver steatosis using histologic assessment as the reference standard.

## Materials and Methods

### Patients

This prospective study was approved by the Institutional Ethics Committee of Pedro Ernesto University Hospital. Written informed consent was obtained from all patients. Between June 2010 and February 2012, type 2 diabetic patients at the University of the State of Rio de Janeiro, Brazil, between 18 and 70 years of age with clinical indications for liver biopsy to evaluate NAFLD were consecutively enrolled. In order to study a homogeneous population with NAFLD, patients with other chronic liver diseases were excluded, because some of them, like hepatitis C can be associated with fatty liver and with fibrosis. Exclusion criteria were: other possible causes of chronic liver disease (positive serology for hepatitis B or C), history of alcoholism (≥20 g of alcohol per day), severe or decompensated cardiopulmonary disease, renal failure (creatinine >1.5 mg/dL), coagulopathy disorders, medication that could cause NAFLD, contraindications to MRI (i.e., claustrophobia or metallic implants), or refusal of liver biopsy. Criteria for withdrawal from the study included other etiology for chronic liver disease upon liver biopsy or insufficient biopsy material for histological analysis.

### MRI and MRS

MRI and MRS were performed during the same examination on a 3.0 T magnet (Philips Medical Systems, Eindhoven, Netherlands) with a Quasar dual gradient system with a peak gradient amplitude of 80 mT/m and slew rate of 200 mT/m/ms. A whole-body transmitter coil and an sixteen-element, receive-only, phased-array coil were used. All patients were supine. Patients suspended respiration at the end of inspiration for breath-hold sequences and were instructed to breathe smoothly for the respiratory-triggered sequences. For correct positioning, coronal localizer images were acquired at maximum inspiration and expiration. Axial and coronal T2 images were obtained for anatomical reference.

### Gradient echo sequences

The liver fat fraction was quantified using T1-weighted, 2D spoiled gradient-echo sequences. Low flip angles were used to minimize T1 effects. To estimate T2* effects, three and seven serial echoes were obtained at different echo times. Sequence parameters are summarized in Table 1.

**Table 1:** Triple- and Multi-echo Sequences Parameters.

	Triple-echo	Multi-echo
TR (ms)/TE (ms)	180/2.3, 3.45, 4.6	180/1.15, 2.3, 3.45, 4.6, 5.75, 6.9, 8.05
Flip angle (degrees)	30	15
Slice thickness (mm)	6	6
Interslice gap (mm)	1	1
Matrix	116×117	116×117
Number of slices	33	33
FOV (mm)	350×350	350×350
NSA	1	1
Acquisition time (s)	43,6	43,6
Number/duration of breath holds (s)	2/21.8	2/21.8
Parallel imaging, acceleration factor	SENSE, 2	SENSE, 2

TR, repetition time; FOV, field of view; NSA, number of signals averaged; SENSE, sensitivity encoding.

### MRS

Single-voxel MRS data were acquired from 30×30×30 mm voxels obliquely positioned by a radiologist (D.B.P. with 10 years of experience with liver imaging) on the Couinaud segment V that corresponded to the location of the biopsy. Localizer sequences at maximum inspiration, maximum expiration, and during free breathing were used to ensure the voxel was inside the liver during the entire respiratory cycle. Care was taken to avoid liver edges, large hepatic vessels, and the biliary tree. Water suppression was not performed and automated shimming generated water line widths of 40–50 Hz.

**Figure 1:**
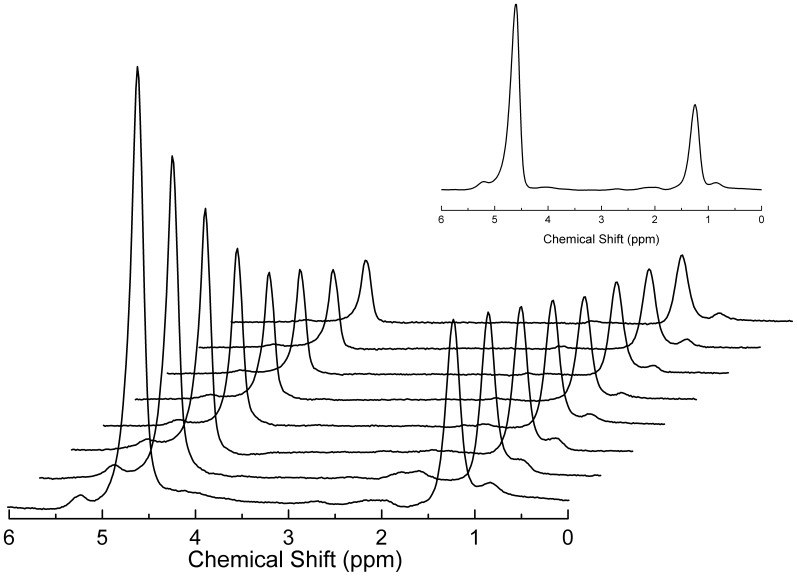
Representative MRS data obtained from a 62-year-old woman with type 2 diabetes and moderate steatosis. T2 estimation was done by fitting the multiecho dataset for both water and fat components considering the spectral modeling. Fat fraction was calculated as described in details throughout the text using the T2-corrected single echo datasets (shown in detail).

Point -resolved spectroscopy sequences (PRESS) were used for MRS acquisition. For T2 correction, a multi-echo version was used and 8 spectra were collected at echo times (TE) of 40, 50, 60, 70, 80, 90, 100, and 110 ms. To minimize T1 effects, the repetition time (TR) was 2000 ms. The number of data points collected was 1024 across a 2000 Hz spectral window and measurements were the averages of 2 acquisitions (Figure 1). To estimate the fat fraction, single-echo, single-voxel MRS data were acquired using the same sequence with the following parameters: a TR of 4000 ms, a TE of 40 ms, a spectral window of 2000 Hz, the number of data points collected was 1024, and the number of acquisitions was 40 (Figure 1 - detail).

### Gradient echo image analysis

**Figure 2:**
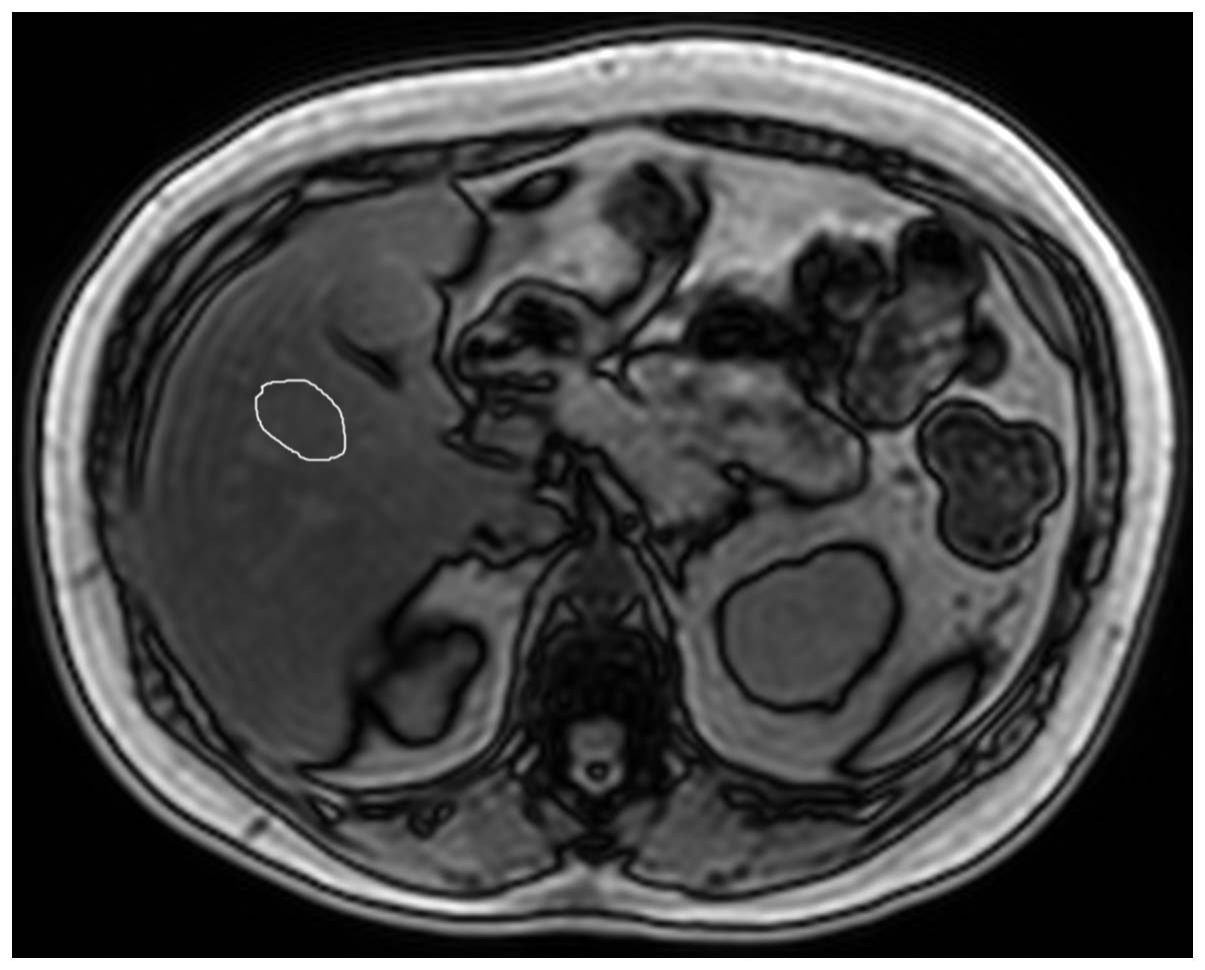
Representative out-of-phase MR images from a 62 year-old woman with type 2 diabetes and moderate steatosis. MR images obtained from breath-hold T1-weighted triple-echo spoiled gradient-echo sequence. ROI was manually drawn at the spectroscopic voxel location (segment V, colocalized with liver biopsy), as shown. ROI, region of interest.

All MR imaging results were interpreted by one radiologist (D.B.P. with 10 years of experience with liver imaging), who was blinded to spectroscopic results. Analysis was performed using a custom analysis package in Matlab (Mathworks, Natick, MA). The radiologist manually drew a region of interest (ROI) approximately 900 mm^2^ in area and placed it at segment V of the liver, in the same region where MRS measurements and biopsies were performed (Figure 2). The fat fraction (including correction for T2* decay and noise bias) was obtained from average ROI values in magnitude images using a multipeak spectral model for fat detection [29], [30].

### MRS analysis

MRS data were analyzed by a MR physicist (F.F.P., 5 years of experience with MRS) who was blinded to imaging results. Analysis used the Advanced Method for Accurate, Robust, and Efficient Spectral (AMARES) algorithm included in jMRUI [31]. At each echo time, the water (4.7 ppm) and fat (0.5–3 ppm) peaks were measured. Each measurable peak area was individually corrected for T2 decay using nonlinear least-square fits to determine their relative proton density. The relative proton densities of the fat peaks located underneath the water peaks were determined according to Hamilton et al. [32]. The total fat proton density was defined as the sum of all T2-corrected individual fat peaks. The proton density fat fraction (PDFF) was calculated as the ratio of the fat proton density to the sum of the fat and water proton densities.

### Liver Biopsy and Evaluation

Liver specimens were obtained from all patients. Subcostal liver biopsies of the segment V of the liver were performed under US guidance using a 16-gauge Menghini biopsy needle. Specimens were 2 cm in length or longer, fixed in 10% formaldehyde solution, and then embedded in paraffin. The sections were then stained with hematoxylin and eosin, Masson's trichrome, and Perls' Prussian blue.

All biopsy slides were examined prospectively by a pathologist (V.L., 28 years of experience). Semiquantitative analysis of steatosis assessed the entire liver fragment and was performed by calculating the percentage of steatotic hepatocytes in the liver parenchyma. Results were classified into the following 4 groups according to the NASH Clinical Research Network (NASH-CRN) scoring system [33]: normal, <5%; mild, 5–33%; moderate, 33–66%; and severe,>66%. Steatosis measurements were scored in increments of 5% for more precise assessment. The pathologist's diagnosis of steatohepatitis was based on the presence of steatosis, hepatocyte injury (ballooning), and lobular inflammation. Fibrosis was scored from F0 to F4 [33]. The presence of siderosis was also evaluated.

### Statistical Analyses

Statistical analyses were performed in R-project (version 2.15). Spearman statistic was used to estimate correlations. Linear regression analyses were conducted to explore the influence of steatohepatitis and fibrosis in liver fat measurements. For the linear models the areas under ROC curve were estimated with the Obuchowski's method[34]. Areas under the ROC curves were estimated by the trapezoidal method for mild (5–33%), moderate (33–66%), and severe steatosis (>66%) as reference values in biopsy evaluations. Decision thresholds were estimated for each MR technique through the maximization of the Youden J index on smoothed robust ROC curves. The ROC curves were compared using a variety of methods with a two-sided test. P values <0.05 indicated significant differences.

## Results

### Patients

**Figure 3:**
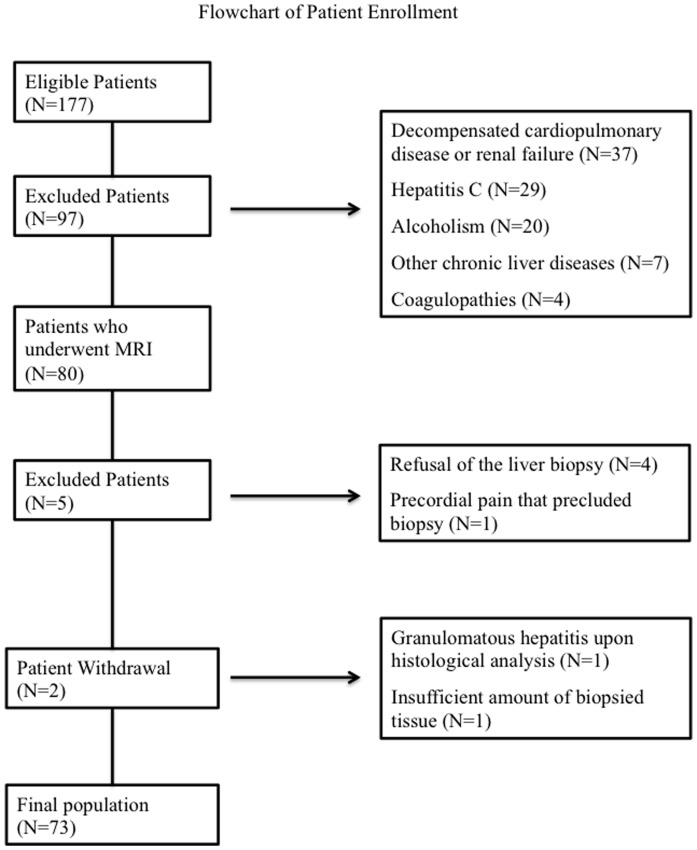
Flowchart of patient enrollment.

A total of 177 patients were interviewed for this study. Ninety-seven patients were excluded: 37 had decompensated cardiopulmonary or renal failure, 29 had hepatitis C, 20 had alcoholism, 7 had other chronic liver diseases, and 4 had coagulopathies. Thus, 80 patients underwent MRI. Five patients were subsequently excluded: 4 refused liver biopsy and 1 had precordial pain that precluded biopsy. Then, two patients were withdrawal: one due to an insufficient amount of biopsied tissue and 1 for granulomatous hepatitis upon histological analysis (Figure 3). Therefore, the final population of the study included 73 patients (60 women [82%] and 13 men [18%]) aged 54±9 years. The mean body mass index (BMI) was 31.4 kg/m^2^ (range, 23.2–42.7 kg/m^2^). Ninety-six percent (70 of 73) of patients were overweight (BMI>25 kg/m^2^) and 62% (45 of 73) were obese (BMI>30 kg/m^2^). MRI and MRS were performed during the same session and within 3 months after the liver biopsy. Thirty-six percent of all patients had elevated aminotransferase levels. Detailed demographic characteristics of the patients are included at Table 2.

**Table 2:** General characteristics of the patients.

	N = 73
Duration of diabetes (years)	11.07±8.12
ALT*	31.59±20.85
AST*	24.66±13.04
Alkaline Fosfatase*	143.19±73.57
Gama-Glutamil transferase*	53.77±41.18
Platelet*	251,616±59,678
Total cholesterol*	188.46±39.10
HDL*	49.28±15.49
Triglycerides*	145.59±69.75
Glucose*	164±63.61
HbA1c levels*	8.59±2.25
Macrovascular complications	5.5%
Microvascular complications	13.7%
Hypertension	75%
Metabolic Syndrome	93.2%
Hypercholesterolemia	45.1%
Statine use	43.1%
Metformine use	90.3%
Insuline use	54.8%

* Mean ± SD.

### Histological Analysis

Upon histology, the prevalence of steatosis (>5%) was 92% (67 patients). Mild steatosis was detected in 35 patients, moderate in 11 patients, and severe in 21 patients. Thirty-seven percent of the patients had steatohepatitis. Fibrosis was observed in 21 (29%) patients. The breakdown of NAFLD fibrosis scores among these patients were: F1, 12 patients; F2, 6 patients; F3, 2 patients; and F4, 1 patient. Two patients had mild siderosis.

### Correlations

**Figure 4:**
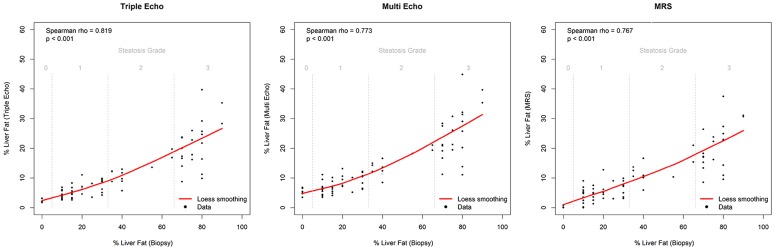
Correlations between triple- and multi-echo sequences and MR spectroscopy versus histopathology examination.

Figure 4 shows correlations of the triple- and multi-echo sequences and MR spectroscopy with histopathology. Both MRI and MRS strongly correlated with liver biopsy findings (triple-echo: rho = 0.819, p<0.001; multi-echo: rho = 0.773, p<0.001; MRS: rho = 0.767, p<0.001). The correlation of PDFF in mild or moderate hepatic steatosis was found to be better than in severe steatosis.

### Diagnostic Accuracy

**Figure 5:**
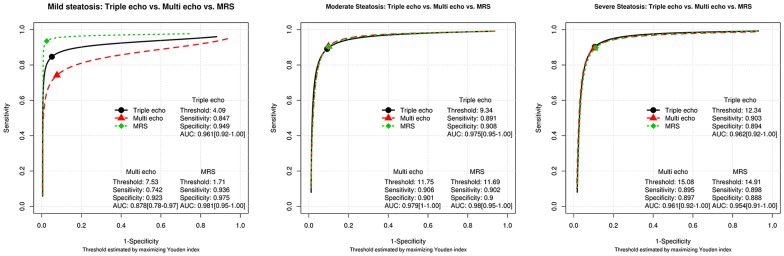
Diagnostic performance for triple- and multi-echo sequences, and MRS using histopathology as the gold standard. The best cut-off point was identified using the Youden index.

The capability of each MR technique to diagnose steatosis was evaluated using histopathology for comparison. The sensitivities, specificities, thresholds, and AUC of each MR technique for mild, moderate, and severe steatosis are shown in figure 5. ROC curves were compared and a significant difference was observed for mild steatosis between multi-echo MRI and MRS (p = 0.033). However, no significant difference between triple-echo and multi-echo MRI (p = 0.093) or between triple-echo MRI and MRS (p = 0.42) was observed for mild steatosis. There was also no significant difference among the different MR methods for the ROC curves in moderate and severe steatosis.

The median fat fraction determined by the different MR methods for mild steatosis varies from 4.98 to 7.06, for moderate steatosis from 10.84 to 14.27, and for severe steatosis from 18.26 to 27.75, and a progressive increase in the interquartile ranges using the same MR method is noted with no overlap (Table 3). Comparative analyses were performed in all groups, analyzed as paired comparisons between two groups to find out the differences between groups. All the comparative analyses showed p<0.05, suggesting that all the groups are different.

**Table 3:** Triple- and Multi-echo MRI and MRS medians for no steatosis, mild, moderate, and severe steatosis using biopsy evaluations as reference values.

	No Steatosis	Mild Steatosis	Moderate Steatosis	Severe Steatosis	Total	p value
	6	35	11	21	73	
Triple-echo median (IQR)	2.62 (2.11,3.08)	5.08 (3.56,7.05)	12.17 (9.32,13.25)	21.75 (16.36,25.68)	8.76 (4.45,16.19)	<0.001
Multi-echo median (IQR)	5.16 (4.77,6.24)	7.06 (5.76,10.12)	14.27 (12.28,17.41)	25.73 (19.48,30.74)	10.69 (6.48,19.14)	<0.001
MRS median (IQR)	0.12 (0.06,0.66)	4.98 (3.12,7.05)	10.84 (10.32,14.53)	18.26 (15.28,24.9)	7.53 (3.59,15.28)	<0.001

MRI, magnetic resonance imaging; MRS, magnetic resonance spectroscopy; IQR, interquartile range;

All the groups were compared by 2×2 independent sample tests and the p values were <0.05 in all the analyses.

One may see that the overall performance measures of the linear models do not change much when a univariable (with imaging only) and multivariable (with steatohepatitis and fibrosis (F0 vs. F1–F4)) are conducted in any of the MR technique (Table 4 and Table 5). Therefore, despite they are significant in the model, fibrosis and steatohepatitis contribute very little to the imaging results.

**Table 4:** Univariable linear regressions analyses for each one of the imaging techniques.

Stats	Estimate	S.E.	Lower 0.95	Upper 0.95
**Triple-echo model**				
Triple-echo	33.829	2.256	29.33	38.327
R2	0.76	-	-	-
ROC AUC	0.865	0.015	-	-
**Multi-echo model**				
Multi-echo	32.891	2.291	28.322	37.46
R2	0.744	-	-	-
ROC AUC	0.848	0.018	-	-
**MRS model**				
MRS	33.318	2.33	28.673	37.963
R2	0.742	-	-	-
ROC AUC	0.845	0.019	-	-

S.E., standard error; ROC, receiver operating characteristic; AUC, area under the curve; MRS, magnetic resonance spectroscopy.

**Table 5:** Multivariable linear regressions analysis for each one of the imaging techniques.

Stats	Effect	S.E.	Lower 0.95	Upper 0.95
**Triple-echo model**				
Triple-echo	28.846	2.43	23.995	33.697
NASH = yes	12.55	4.056	4.453	20.647
fibrosis = 1	−0.512	4.31	−9.115	8.09
fibrosis = 2	16.376	6.004	4.391	28.361
fibrosis = 3 or 4	−5.499	7.306	−20.083	9.084
R2	0.841	-	-	-
ROC AUC	0.88	0.014	-	-
**Multi-echo model**				
Multi-echo	27.718	2.4	22.927	32.508
NASH = yes	14.533	4.076	6.398	22.668
fibrosis = 1	−1.147	4.412	−9.954	7.66
fibrosis = 2	15.11	6.114	2.906	27.315
fibrosis = 3 or 4	−7.03	7.426	−21.851	7.792
R2	0.835	-	-	-
ROC AUC	0.864	0.016	-	-
**MRS model**				
MRS	27.248	2.444	22.371	32.125
NASH = yes	14.645	4.173	6.316	22.975
fibrosis = 1	1.143	4.455	−7.749	10.036
fibrosis = 2	13.483	6.258	0.992	25.975
fibrosis = 3 or 4	−8.063	7.589	−23.212	7.085
R2	0.828	-	-	-
ROC AUC	0.864	0.016	-	-

S.E., standard error; ROC, receiver operating characteristic; AUC, area under the curve; NASH, non-alcoholic steatohepatitis; MRS, magnetic resonance spectroscopy.

## Discussion

Our study evaluated two MRI chemical shift techniques (triple- and multi-echo imaging) and MRS to diagnose steatosis and quantify fat in the livers of patients with type 2 diabetes. We compared these techniques to histopathological findings. Our results demonstrated that all MR techniques had high sensitivity and specificity for NAFLD diagnosis and strongly correlated with histopathology. In mild steatosis, a similar performance was observed between triple-echo sequences and MRS. In moderate and severe steatosis there was no significant difference among triple- and multi-echo sequences and MRS.

Although steatosis measurements by MR and histology were correlated, the actual values were different because the parameters used to estimate liver fat are different. Pathologists quantify the percentage of hepatocytes containing fat, whereas MR measures the signal from fat protons. Idilman et al. [35] found that PDFF better correlated with mild or moderate hepatic steatosis than severe steatosis, which is consistent with our data (Figure 4). These results suggest that hepatocytes in severe steatosis contain varied quantities of fat, but are not distinguished from each other upon histopathology (each is counted as a fat-containing hepatocyte). However, MR methods quantify the amount of fat in tissue. The fat fraction can be>2 times greater by histopathological assessment than by MR techniques, suggesting that many hepatocytes contain little fat. However, MRI and MRS measurements are similar [36], [37] to each other. Studies with larger numbers of patients should be performed to better understand the relationship between fat measurements by MR and histopathology.

Few studies have evaluated the influence of steatohepatitis or fibrosis in liver fat quantification [35], [38]. Although Idilman et al. [45]. found that hepatic fibrosis reduced the correlation between biopsy results and liver fat fraction, our results are similar to the ones of Tang et al. [38], where the presence of steatohepatitis and/or fibrosis did not affect MR steatosis measurements.

Our threshold values for the detection of steatosis were similar to those in other studies that used histopathology [36], [37], [39]–[42], but the appropriate thresholds have yet to be defined. In our study, the threshold for the detection of mild steatosis varied from 1.71 using MRS, to 4.09 using triple-echo sequence, to 7.53 using multi-echo MRI. Only a small overlap was observed between patients with no steatosis vs. mild steatosis for multi-echo sequence, where the IQR 75 for no steatosis was 6.24 and the IQR 25 for mild steatosis was 5.76. Neither other MR method nor other group had any overlap, suggesting that the technique may be able to accurately differentiate the patient groups. In addition, there were proportional increasing fat fraction medians for all MR techniques in patients with higher steatosis grades determined by histopathology. Thus, we believe that triple-echo, multi-echo, and MR spectroscopy are accurate non-invasive imaging methods for the diagnosis, grading, and follow-up of NAFLD.

Hepatic siderosis distorts the local magnetic field and causes loss of phase coherence and T2* shortening, which leads to signal loss at in-phase times compared to out-of-phase times [43], [44]. The T2* relaxation time varies widely among patients and using theoretical T2* relaxation times may lead to incorrect interpretations [45]. The T2* relaxation time can be calculated for each patient using triple- and multi-echo MRI, and with T2* correction, may prevent misinterpretation in cases of hepatic siderosis, a condition that can be associated with NAFLD [46]. In this study, only 2 patients had hepatic siderosis. Thus, we could not evaluate its effects on liver fat quantification.

Gradient-echo MRI appears to be the most appropriate for routine clinical use. In moderate and severe steatosis, no significant difference was observed for all MR methods. In our study, triple-echo sequence showed excellent correlation to histopathology and was similar to MRS to detect and quantify the liver fat fraction in mild steatosis. In addition, gradient-echo MR imaging can measure the liver fat fraction throughout the liver, has a short acquisition time, can be performed as a breath-hold sequence, and does not require hardware upgrades. Furthermore, it allows individual T2* corrections and the post-processing is easier, faster, and less prone to misinterpretation than MRS.

Although MRS enables highly accurate liver fat quantification, it requires strong magnetic fields, data processing is more complex and time consuming, it requires a large team highly specialized personnel, and is much more prone to errors. Lastly, it evaluates only a single voxel, which can lead to misinterpretation in cases of heterogeneous steatosis.

This study has some limitations. First, MRS was performed using a PRESS sequence, which may be more sensitive to J coupling effects [47]. This could lead to a systematic underestimation of fat relaxation times and slightly overestimate PDFF. Also, data acquisition time could be reduced by employing a single signal acquisition. We averaged many MRS acquisitions to ensure high signal-to-noise ratios and minimize baseline variations. Therefore, MRS had to be performed during free breathing where voxels moved 2–3 cm in the longitudinal plane. To minimize motion-related bias, the protocol included a coronal sequence during maximal inspiration and another during maximal expiration to ensure voxels remained properly positioned in the liver during acquisition. The MRI and MRS analyses were performed only in segment V, in the location corresponding to the liver biopsy and other liver segments were not evaluated to maintain clinically reasonable scan times. MRS voxels could also be difficult to position because of the biliary tree, vessels, and magnetic susceptibility in other liver segments. The prevalence of NAFLD in our population was extremely high and this could have negatively influenced the specificity, but this is inherent in the studied population. To the best of our knowledge, this is the first study to compare fat quantification by MRI and histopathology in diabetic patients. In addition, our study evaluated the performance of triple- and multi-echo MRI and MRS, with individual T2* and T2 corrections, respectively, which are important steps to allow comparisons between different magnets at different institutions.

In summary, MR measurements may be useful to screen for NAFLD. Liver fat quantification by MRI and MRS strongly correlated with histopathology, even in the presence of steatohepatitis and fibrosis. Triple- and multi-echo MRI and MRS were highly accurate and may be used as a substitute for liver biopsy. Due to the wide availability and easier post-processing, gradient-echo MRI appears to be the most appropriate for routine clinical use in the evaluation of NAFLD in diabetic patients.
